# Psychological distress in victims of intimate partner violence: the buffering effect of post-traumatic growth and metacognitive abilities

**DOI:** 10.3389/fpsyg.2026.1722892

**Published:** 2026-04-08

**Authors:** Rossella Procaccia, Giulia Segre, Marco Castiglioni, Francesco Rotunno, Cristina Liviana Caldiroli

**Affiliations:** 1 Faculty of Psychology, Department of Theoretical and Applied Sciences (DiSTA), eCampus University, Novedrate, Italy; 2 Department of Human Sciences for Education “Riccardo Massa”, University of Milano-Bicocca, Milan, Italy; 3 Department of Sociology and Social Research, University of Milano-Bicocca, Milan, Italy

**Keywords:** depression, intimate partner violence (IPV), metacognition, post-traumatic growth (PTG), post-traumatic stress disorder (PTSD), psychopathology

## Abstract

Intimate partner violence (IPV) is a pervasive public health issue associated with severe psychological consequences, including post-traumatic stress disorder (PTSD), depression, and global psychopathology. Nevertheless, many survivors also experience positive psychological changes, defined as post-traumatic growth (PTG). The present study aimed to examine the associations between psychopathological symptoms, metacognitive abilities, and post-traumatic growth, and to test whether metacognitive abilities mediate the relationship between psychological distress and PTG in women survivors of IPV. In a cross-sectional design, thirty-one women survivors of IPV (*M* = 42.77 years; SD = 11.93) were recruited through specialized support services in Northern and Central Italy. Participants completed standardized self-report measures assessing PTSD symptoms, depression, global psychopathology, post-traumatic growth, and metacognitive abilities. Mediation models were tested using correlational and hierarchical multiple regression analyses with bootstrapping procedures. Results showed that PTSD, depression, and global psychopathology were negatively correlated with PTG and metacognitive abilities, whereas metacognition positively correlated with PTG. Regression analyses revealed that metacognition fully mediated the effects of PTSD and global psychopathology on PTG and partially mediated the effect of depression. Higher metacognitive abilities were associated with greater post-traumatic growth despite elevated symptom levels. Findings suggest that metacognitive abilities act as a key intrapersonal resource in trauma recovery, facilitating emotional regulation, cognitive processing, and meaning-making. Enhancing metacognitive functioning could strengthen therapeutic interventions for IPV survivors by promoting adaptive coping and growth beyond symptom reduction.

## Introduction

Intimate partner violence (IPV) refers to any behavior by a current or former partner that results in physical, sexual, or psychological harm, including aggression, coercion, emotional abuse, and economic abuse (Ahmad et al., 2017; Stylianou, 2018; Benfante et al., 2024). It is typically characterized by patterns of control and coercion exerted by one partner over the other and represents a major clinical and public health issue worldwide (Peterson et al., 2024; White et al., 2024a). IPV occurs across all settings and within all socioeconomic, religious, and cultural contexts (Bacchus et al., 2024). Evidence further indicates that IPV tends to be transmitted inter-generationally: women whose mothers were abused are approximately twice as likely to experience violence themselves compared to women whose mothers were not abused (Keynejad and Howard, 2025). According to the United Nations Women (2021), intimate partner violence is recognized as a form of gender-based violence that disproportionately affects women worldwide, who continue to bear the greatest global burden of this form of abuse. Global prevalence estimates suggest that 30%–40% of women aged 15 years and older have experienced physical, psychological, or sexual violence from an intimate partner during their lifetime (World Health Organization, 2021; CDC, 2022; White et al., 2024a). A meta-analysis further estimated that nearly 40% of female homicides are perpetrated by intimate partners (Wang, 2025). According to the most recent estimates (ISTAT, 2025), approximately 6.4 million Italian women (31.9%) aged 16–75 have experienced at least one episode of physical or sexual violence during their lifetime; 18.8% have suffered physical violence and 23.4% sexual violence, including rape or attempted rape (5.7%). Among women with a current or former partner, 12.6% report physical or sexual violence within the relationship, alongside psychological (17.9%) and economic abuse (6.6%) perpetrated by partners or ex-partners. IPV entails not only profound individual suffering but also substantial societal and economic costs (Vyas et al., 2023).

The health consequences of IPV are severe and widespread, encompassing both immediate and long-term sequelae (Lortkipanidze et al., 2025). Victims face an increased risk of injury (Liu et al., 2020; de Souza et al., 2024), chronic pain (Pierce et al., 2026), headaches and migraines (Campbell et al., 2018), as well as gastrointestinal and gynecological problems (Hutchinson et al., 2023; Pallotta et al., 2024). Beyond physical outcomes, a strong bidirectional relationship exists between IPV and mental health: IPV predicts psychological difficulties, and pre-existing mental health vulnerabilities increase the risk of IPV exposure (Bacchus et al., 2024; Procaccia and Castiglioni, 2022).

From a psychological perspective, post-traumatic stress disorder (PTSD) is the most frequently reported outcome among women survivors of IPV, with prevalence rates ranging from 31 to 84% (Cannon et al., 2024; Ragucci et al., 2024). Meta-analytic evidence indicates that IPV significantly increases the risk of depression, anxiety, and suicidality (Kandeğer and Naziroğlu, 2021; Cirici Amell et al., 2023; Kim, 2023; White et al., 2024a; Naismith et al., 2024; Torrioni et al., 2025). Other common outcomes include sleep disturbances (Maharaj et al., 2021; Mueller et al., 2025), substance use (Gibbs et al., 2018; Ogum Alangea et al., 2018), stigma (Taccini and Mannarini, 2023; Murvartian et al., 2024), and, in extreme cases, homicide (Kim, 2023).

Emotional responses to IPV include both negative reactions, such as self-blame and anger, and positive emotions, including hope and acceptance (Sere et al., 2021). Consequently, the recovery process is characterized by the coexistence of suffering and growth, resilience and psychopathology (Fernández Álvarez et al., 2022; Castiglioni et al., 2023a). While much research has focused on the negative consequences of IPV, fewer studies have examined processes of healing and recovery. Survivors often draw on personal and social resources, and many reports positive learnings from their traumatic experiences, such as engagement in activism or helping other women (Bryngeirsdottir et al., 2022; Crapolicchio et al., 2023; Mantler et al., 2024).

Many of these positive reactions are captured by the construct of post-traumatic growth (PTG), defined as “positive psychological change experienced as a result of the struggle with highly challenging circumstances” (Tedeschi and Calhoun, 2004). The literature suggests that trauma may act as a catalyst for PTG (Ulloa et al., 2015; Castiglioni et al., 2023b). Importantly, focusing on positive post-trauma changes does not minimize the adverse effects of trauma. Rather, PTG is thought to emerge through processes of meaning-making following experiences that initially generate high levels of psychological distress (Tedeschi and Calhoun, 2004). Research has increasingly examined post-traumatic growth (PTG) in women who have experienced intimate partner violence, showing that survivors may experience meaningful psychological and personal development despite the trauma. (Bakaitytė et al., 2022; Bryngeirsdottir and Halldorsdottir, 2022a; Machinga-Asaolu, 2024).

Several studies have examined the role of metacognitive abilities in trauma processing. Metacognition refers to the capacity to understand and interpret one’s own and others’ behaviors in terms of mental states, as well as to use this understanding to regulate distress and solve problems. An operationalized model distinguishes separable domains, including understanding one’s own mind, understanding others’ minds, and mastery (Semerari et al., 2003; Lysaker and Dimaggio, 2014). More broadly, metacognitive abilities encompass strategies used to reflect on and regulate one’s own thinking and learning processes, such as planning task approaches, monitoring progress and comprehension, and evaluating outcomes. Through the development of metacognitive abilities, individuals may enhance self-awareness, goal-setting, strategic flexibility, and self-evaluation.

Emerging evidence indicates that metacognitive functioning may serve as a protective factor across both clinical and non-clinical populations. Higher metacognitive capacity has been linked to lower levels of psychopathology and greater resilience in general populations (Giugliano et al., 2022; Sánchez Marqueses et al., 2022). Specifically, Giugliano et al. (2022) found that stronger metacognitive abilities predict fewer psychotic experiences, whereas Sánchez Marqueses et al. (2022) reported that post-traumatic growth is associated with metacognitive beliefs, self-focused attention, and trauma-related attitudes, with trauma exposure and post-traumatic stress symptoms emerging as the most robust predictors.

Recent research highlights the role of metacognitive strategies in emotional regulation following trauma and their association with PTG (Li et al., 2020; Brown et al., 2022; Bryngeirsdottir et al., 2022). In particular, Bryngeirsdottir et al. (2022) proposed an eight-component theoretical model identifying facilitating factors of PTG, including recognition of harm, social support, activation, and cognitive elaboration of the traumatic experience. In women survivors of IPV, metacognitive abilities have been linked to adaptive outcomes and shown to buffer the effects of pathological affective dependence on psychological functioning (Pugliese et al., 2025). Conversely, deficits in metacognition are associated with an increased risk of aggression, whereas higher metacognitive skills appear to protect both victims and perpetrators of violence (Misso et al., 2019; Candini et al., 2020). Moreover, metacognitive processes have been found to mediate symptom reduction in IPV survivors (Procaccia and Castiglioni, 2022) and to play a central role in PTSD recovery (Wells and Sembi, 2004).

Although the literature has highlighted the role of cognitive abilities in trauma processing, the precise mechanisms through which these abilities influence recovery remain insufficiently understood. In particular, while cognitive and emotional factors have been extensively examined in relation to posttraumatic stress and psychological adjustment, the specific contribution of metacognitive functioning has received comparatively limited attention. Understanding how metacognitive abilities may buffer the impact of psychopathological symptoms and facilitate adaptive outcomes is especially relevant, as these skills represent potentially modifiable targets for trauma-focused interventions.

For this reason, the present study aimed to examine both the psychopathological consequences of intimate partner violence (IPV) and the potential mediating role of metacognitive abilities in shaping the relationship between psychopathology and post-traumatic growth (PTG).

Specifically, we hypothesized that:

*(H1)*: Women survivors of IPV would report high levels of psychological distress (PTSD, depression, and global psychopathology) as well as positive resources, including PTG and metacognitive abilities.

*(H2)*: Higher levels of PTSD, depression, and global psychopathology would be associated with lower levels of post-traumatic growth.

*(H3)*: Higher metacognitive abilities would be associated with greater post-traumatic growth.

*(H4)*: Metacognitive abilities would mediate the effects of PTSD, depression, and global psychopathology on post-traumatic growth.

## Materials and methods

### Participants

Thirty-one women who were victims of intimate partner violence (IPV) (mean age = 42.77 years, SD = 11.93) participated in the present study. Participants were recruited through services for abused women located in Northern and Central Italy. Inclusion criteria were: (1) having experienced IPV; (2) being at least 18 years old; (3) sufficient proficiency in written and spoken Italian; and (4) currently living in safe conditions, defined as complete separation from the abusive partner for at least 30 days. Most participants were Italian, had a medium level of education, and were currently employed. The majority had been married to or in a stable cohabiting relationship with their partners. Participants reported chronic exposure to multiple forms of abuse, and most had been separated from the abusive partner for less than 6 months (see Table 1).

**Table 1:** Demographic.

	Data	Indicator
*Age (years)*		
Mean (SD)	42.77 (11.93)	
Min–max	21	60
*Nationality*		
Italian	25	80.6%
Not Italian	6	19.4%
*Education*		
Middle school license	16	51.6%
Degree	10	32.3%
Post-graduate degree	5	16.1%
*Occupational status*		
Employed	19	61.3%
Unemployed	12	38.7%
*Marital status*		
Married or cohabiting	20	64.51%
Stable partner	11	35.49%
*Children*		
Children	29	93.54%
No children	2	6.46%
Number of children	Min–max: 0–5	Mean = 1.08 DS = 1.13
*Year of victimization*		
<1 year	6	19.35%
>1 year	25	80.65%
*Separation from abusing partner*		
<6 months	19	61.3%
>6 months	12	38.7%
*Type of victimization*		
Psychological abuse	31	100%
Physical abuse	27	87.09%
Sexual abuse	26	83.87%
More than one type of abuse	31	100%

### Measures

#### Demographic characteristics

Participants reported their age, ethnic background, level of education, number of children, marital or relationship status, duration of victimization, and type of abuse experienced (sexual, physical, and psychological).

#### Beck depression inventory-II (BDI-II)

The Beck depression inventory-II (BDI-II; Beck et al., 1996a,b; Italian validation by Ghisi et al., 2006) was used to assess depressive symptoms. The BDI-II is a 21item self-report measure evaluating cognitive, affective, motivational, and behavioral components of depression. Items are rated on a 4-point Likert scale ranging from 0 (never) to 3 (always), yielding a maximum total score of 63. Based on the Italian validation study, a cutoff score greater than 12 was used to indicate the presence of depressive symptoms. Scores from 13 to 19 indicate minimal depression, scores from 20 to 28 indicate moderate depression, and scores from 29 to 63 indicate severe depression. Previous studies have reported good internal consistency, with Cronbach’s *α* ranging from 0.80 to 0.87 in normative and clinical samples (Lee et al., 2017). In the present study, Cronbach’s *α* was 0.83.

#### Los Angeles symptom checklist (LASC)

The Los Angeles symptom checklist (LASC; King et al., 1995) was administered to assess posttraumatic stress disorder (PTSD) symptoms. This 43-item self-report instrument measures global distress related to trauma exposure, overall PTSD severity, and the severity of specific PTSD symptom clusters (i.e., re-experiencing, avoidance/numbing, and hyperarousal). Previous research has demonstrated high internal consistency, with Cronbach’s *α* coefficients ranging from 0.88 to 0.95 (Foy, 1993). In the present study, Cronbach’s *α* was 0.89. The LASC items were translated into Italian using a back-translation procedure.

#### Symptom checklist-90-revised (SCL-90-R)

Global psychopathological symptomatology was assessed using the symptom checklist-90-revised (SCL-90-R; Derogatis, 1994; Italian version by Prunas et al., 2012). The SCL-90-R is a 90-item self-report questionnaire assessing psychological symptoms experienced during the previous week. Items are rated on a 5-point Likert scale ranging from 0 (not at all) to 4 (extremely). The instrument comprises 10 primary symptom dimensions: somatization, obsessive-compulsive symptoms, interpersonal sensitivity, depression, anxiety, hostility, phobic anxiety, paranoid ideation, psychoticism, and sleep disturbances. For each dimension, scores are calculated as the mean of the corresponding items. A Global Severity Index (GSI) is also computed as the mean of all items, representing overall psychological distress. Cronbach’s *α* coefficients above 0.70 are considered acceptable (Peterson, 1994). In the present study, Cronbach’s *α* was 0.95.

#### Posttraumatic growth inventory (PTGI)

The Posttraumatic Growth Inventory (PTGI; Tedeschi and Calhoun, 1996; Italian adaptation by Prati and Pietrantoni, 2014) was used to assess positive psychological changes following trauma. The PTGI is a 21-item self-report measure assessing perceived positive outcomes resulting from traumatic experiences. Participants were instructed to rate the degree of change in their lives resulting from their experience in an abusive relationship. The PTGI comprises five factors: New Possibilities, Relating to Others, Personal Strength, Spiritual Change, and Appreciation of Life. Items are rated on a Likert scale ranging from 0 (I did not experience this change as a result of my crisis) to 5 (I experienced this change to a very great degree as a result of my crisis). The PTGI has demonstrated good construct validity and acceptable test-retest reliability over a 2-month interval (*r* = 0.71). In the present sample, internal consistency was excellent (*α* = 0.95). Subscale reliabilities were also adequate: New Possibilities (*α* = 0.85), Relating to others (*α* = 0.88), personal strength (*α* = 0.83), spiritual change (*α* = 0.84), and appreciation of life (*α* = 0.77).

#### Metacognition Rating Scale (SVaM)

Metacognitive abilities were assessed using the Metacognition Rating Scale (SVaM; Carcione et al., 1997; English version by Semerari et al., 2003). This instrument evaluates metacognitive functions as expressed in participants’ verbalizations. The scale comprises three domains: (a) Self-reflexivity, referring to the ability to reflect on one’s own mental states; (b) Understanding the minds of others (decentralization), referring to the ability to reflect on others’ mental states; and (c) Mastery, referring to the use of psychological knowledge to regulate distress, solve interpersonal problems, and develop adaptive coping strategies. In the present study, Cronbach’s *α* for the total scale was 0.81.

### Procedure

All participants completed an informed consent form describing the aims of the study, a sociodemographic questionnaire, and the full battery of research instruments. The consent form highlighted the potential risks associated with participation, including emotional distress related to recalling traumatic experiences, and informed participants of their right to withdraw from the study at any time without consequences. Participants completed the questionnaires individually at home. In addition, they were asked to complete a written narrative task focused on the positive and negative changes they perceived following their abusive experiences. All narratives were analyzed using the SVaM by two independent judges, with an interrater agreement of approximately 0.80. Data were collected between February and June 2025. The study was conducted in accordance with the Ethics Code of the Italian Psychological Association and was approved by the Ethics Committee of eCampus University (No. 08/2024). Participants’ personal data were processed in compliance with the General Data Protection Regulation (GDPR; EU Regulation 2016/679).

### Statistical analyses

To test the first hypothesis (H1), descriptive analyses were conducted to compute participants’ baseline levels of posttraumatic stress symptoms (PTSD), depression, global psychopathology, posttraumatic growth (PTG), and metacognitive abilities. To test (H2) and (H3), Pearson’s r correlation analyses were then performed to examine the associations among these variables. To test the mediational hypothesis (H4), a series of hierarchical multiple regression analyses was conducted following the procedure outlined by Baron and Kenny (1986). Three separate mediation models were tested, with depression, PTSD, and global psychopathology, respectively, entered as predictors, posttraumatic growth as the outcome variable, and metacognitive abilities as the mediator. According to the Baron and Kenny framework, mediation was evaluated by verifying four conditions: (1) the predictor (depression, PTSD, or global psychopathology) was significantly associated with the outcome variable (PTG); (2) the predictor was significantly associated with the mediator (metacognitive abilities); (3) the mediator was significantly associated with the outcome variable when controlling for the predictor; and (4) the association between the predictor and the outcome was reduced (partial mediation) or became non-significant (full mediation) when the mediator was included in the model. Indirect effects were further tested using a bootstrapping procedure with 1,000 resamples and 95% bias-corrected confidence intervals. All statistical analyses were conducted using SPSS version 21.

## Results

### Psychological distress and positive resources in survivors of IPV

Descriptives showed that participants suffered severe PTSD symptoms (LASC cut-off values by King et al., 1995), high level of global psychopathology (SCL-90-R; Derogatis, 1994; Italian version by Prunas et al., 2012) and more than half (58.1%) presented moderate or severe depression symptoms (BDI-II cut off by Beck et al., 1996a,b). Nevertheless, medium-high levels of post traumatic growth (Tedeschi and Calhoun, 1996) and metacognitive abilities were found (Carcione et al., 1997) (see Table 2).

**Table 2:** Descriptive analysis.

Variable	Min	Max	Mean	SD
PTSD	6.00	71.00	36.10	17.33
Depression	0.00	81.00	23.35	19.22
Global psychopathology	10.00	199.00	114.65	55.40
PTG	22.00	85.00	58.06	17.51
Metacognitive abilities	17.00	69.00	45.68	15.15
Depression (categorical values)	None	Low	Moderate	Severe
*n* (%)	8 (25.8%)	7 (22.6%)	8 (25.8%)	8 (25.8%)

### Associations between PTSD, depression, global psychopathology, post traumatic growth and metacognition

The correlational analyses (see Table 3) showed that both PTSD, depression and global psychopathology were negatively correlated with post traumatic growth and metacognition abilities. Furthermore, post traumatic growth and metacognition abilities were positively correlated.

**Table 3:** Correlational analysis.

Variable	PTGI	PTSD	Depression	Global psychopathology	Metacognitive ability
1. PTGI	1	−0.650^**^	−0.610^**^	−0.424^*^	0.685^**^
2. PTSD	−0.650^**^	1	0.768^**^	0.726^**^	−0.730^**^
3. Depression	−0.610^**^	0.768^**^	1	0.582^**^	−0.562^**^
4. Global psychopathology	−0.424^*^	0.726^**^	0.582^**^	1	−0.586^**^
5. Metacognitive ability	0.685^**^	−0.730^**^	−0.562^**^	−0.586^**^	1

^**^p < 0.01; ^*^p < 0.05.

### Mediational models

**Figure 1:**
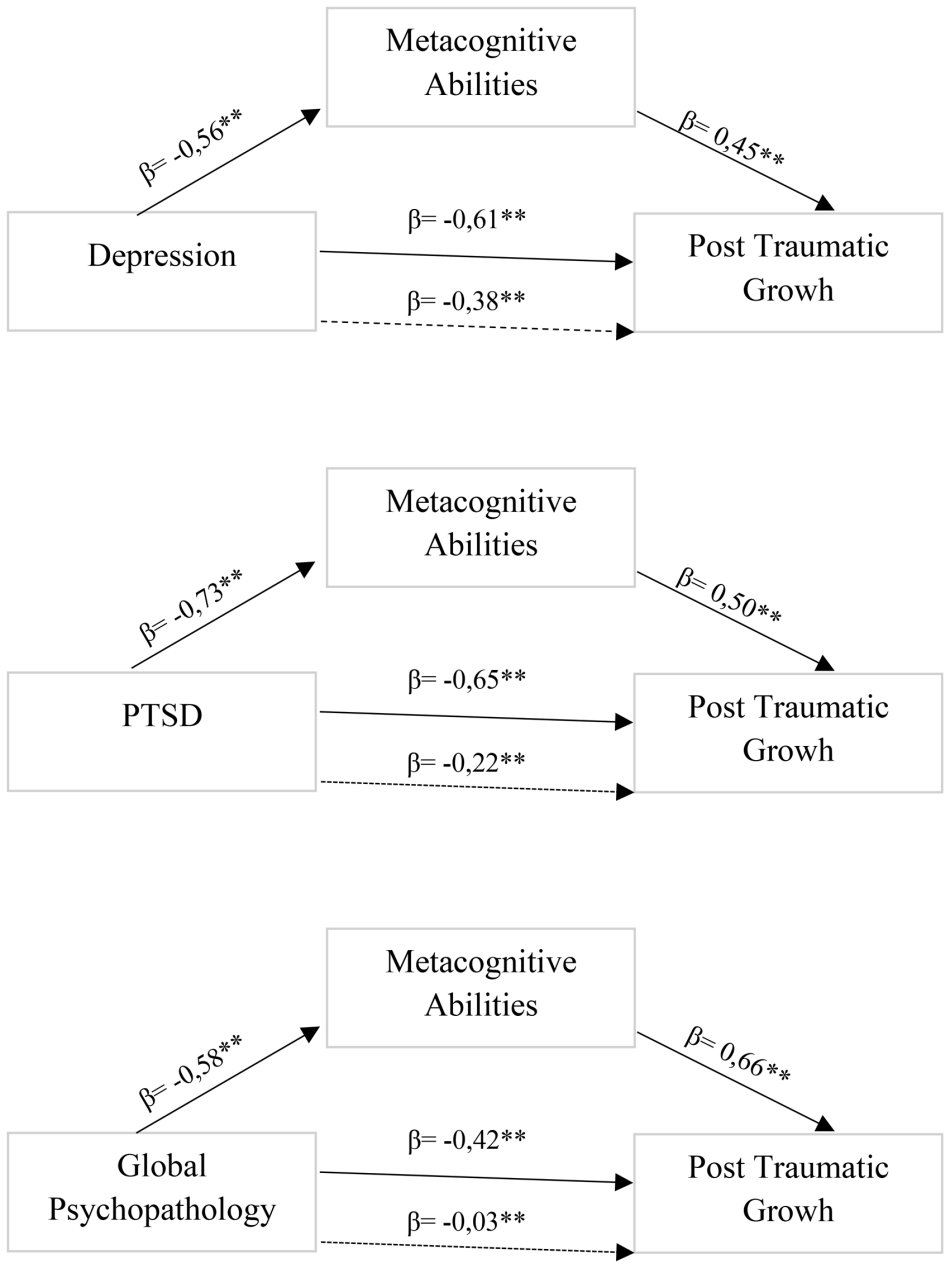
Mediational models.

We performed multiple regression analyses, with post traumatic growth score as the dependent variables, depression, PTSD and global psychopathology as predictors, and metacognition abilities as mediator, finding that (see Figure 1):

Low post traumatic growth was predicted firstly by high PTSD score (*β* = −0.65; *t* = −4.60, *p* = 0.001), secondarily by high depression score (*β* = −0.61; *t* = −4.14, *p* = 0.001) and finally by high global psychopathology score (*β* = −0.42; *t* = −2.51, *p* = 0.01). Metacognition abilities fully mediated PTSD effects (PTSD: *β* = −0.22; *t* = −2.13, *p* = 1.05; Metacognition: *β* = 0.50; *t* = 3.23, *p* = 0.003) and global psychopathology effects (Global psychopathology: *β* = −0.03; *t* = −0.20, *p* = 0.84; Metacognition: *β* = 0.66; *t* = 3.91, *p* = 0.001), and partially mediated depression effects (Depression: *β* = −0.38; *t* = −1.87, *p* = 0.02); Metacognition: *β* = 0.45; *t* = 2.34, *p* = 0.02).

## Discussion

The present study investigated the psychopathological sequelae of intimate partner violence (IPV) and examined the potential mediating role of metacognitive abilities in the association between psychological distress and post-traumatic growth (PTG). Consistent with our hypotheses, we expected women survivors of IPV to report elevated psychological distress (PTSD, depressive symptoms, and global psychopathology) alongside positive resources, including PTG and metacognitive abilities. We further hypothesized that distress would be negatively associated with PTG, whereas metacognitive abilities would be positively related to growth and would mediate the relationship between psychological distress and post-traumatic growth.

In line with our first hypothesis (H1) and prior empirical evidence (White et al., 2024b; Lortkipanidze et al., 2025), women survivors of IPV reported heightened levels of psychological distress, including PTSD symptoms, depressive symptomatology, and global psychopathology. At the same time, they also endorsed the presence of positive psychological resources, such as post-traumatic growth and metacognitive abilities (Bryngeirsdottir and Halldorsdottir, 2022b).

In line with Hypothesis 2, we found that higher levels of PTSD symptoms, depressive symptoms, and global psychopathology were significantly associated with lower posttraumatic growth, indicating that greater psychological distress is related to reduced positive adjustment following IPV. Metacognitive abilities showed a positive association with posttraumatic growth, supporting the notion that the capacity to reflect on, monitor, and regulate one’s mental states may foster adaptive meaning-making after trauma (H3). With respect to Hypothesis 4, mediation analyses identified metacognitive abilities as a key mediator in the relationship between psychological distress and posttraumatic growth. Specifically, our results showed that metacognition fully mediated the effects of PTSD symptoms and global psychopathology on posttraumatic growth and partially mediated the association between depressive symptoms and growth. These findings suggest that the negative impact of trauma-related distress on posttraumatic growth may largely operate through disruptions in metacognitive functioning. Accordingly, metacognition may represent a crucial intrapersonal resource supporting trauma processing, reflective functioning, identity reorganization, and cognitive elaboration—processes central to theoretical models of posttraumatic growth.

This pattern of findings is consistent with cognitive and metacognitive theories of trauma, as well as with emerging literature highlighting the role of metacognitive beliefs, attentional control, and cognitive regulation processes in shaping not only psychopathological outcomes but also the ways individuals process trauma and potentially achieve growth. According to the Self-Regulatory Executive Function (SREF) model (Wells and Matthews, 1996), psychological distress is maintained not solely by cognitive content (e.g., negative beliefs about the self, the trauma, or the world), but primarily by dysfunctional metacognitions—such as beliefs about the uncontrollability of thoughts, persistent rumination and worry, and difficulties disengaging from threat-focused attention—along with the associated cognitive attentional syndrome.

The present findings, showing that metacognitive abilities mediate the effects of PTSD and general psychopathological symptoms on posttraumatic growth, are consistent with this model: when individuals possess stronger metacognitive skills, the negative impact of PTSD and other symptoms on PTG is attenuated or even eliminated. Recent research (Agnoli et al., 2024) supports the view that dysfunctional metacognitive processes are closely associated not only with symptom severity but also with the maintenance of PTSD. The observation that mediation was complete for PTSD and global psychopathology, but only partial for depression, suggests that metacognitive skills may be particularly effective in interrupting the core maintaining mechanisms of PTSD—such as intrusions, hyperarousal, avoidance, and trauma-related rumination—whereas depression may involve additional processes not fully captured by metacognitive functioning alone. These may include neurobiological alterations, reduced motivation, anhedonia, and deficits in behavioral or social activation.

Importantly, posttraumatic growth is not merely the absence of symptoms. Rather, it requires active processes of reflection, cognitive restructuring, and personal meaning-making. Metacognitive abilities appear to facilitate these processes by enabling more flexible self-reflection, improved regulation of emotions and intrusive thoughts, and the modulation of self-focused attention through adaptive strategies (e.g., reducing rumination and increasing awareness of thought patterns). In the absence of adequate metacognitive skills, severe psychological symptoms may overwhelm available resources and hinder the possibility of growth.

### Clinical implications

The findings have several clinical implications for intervention programs targeting women survivors of IPV. First, they underscore the importance of assessing metacognitive functioning early in the therapeutic process. Beyond evaluating levels of PTSD, depression, or general psychopathology, it may be clinically valuable to assess metacognitive beliefs, monitoring and control abilities, rumination management, and metacognitive coping strategies. Second, the results highlight the potential benefit of integrating interventions aimed at enhancing metacognitive skills to promote posttraumatic growth. For example, Metacognitive Therapy (MCT; Wells, 2009), or selected metacognitive techniques, could be used to modify dysfunctional beliefs (e.g., “I cannot control my thoughts,” “Thinking about the trauma will destroy me”), promote distance from intrusive thoughts, reduce maladaptive rumination, and foster more adaptive metacognitive strategies.

At the same time, the partial mediation observed for depression suggests the need for tailored interventions. Treatment for depressive symptoms may require additional components, such as psychoeducation, behavioral activation, interventions promoting social engagement, self-efficacy, self-compassion, and interpersonal support, alongside metacognitive work. Furthermore, it is essential to recognize that metacognitive skills do not operate in isolation. In IPV contexts, ongoing threats, limited social support, stigma, and scarce resources may compromise the effective use of these skills. Interventions should therefore address both individual-level processes and broader environmental and contextual factors.

### Limitations and future directions

Despite these promising findings, several limitations should be acknowledged. First, the relatively small sample size limits the generalizability of the results, and the findings should be considered exploratory. Although bootstrapping procedures were employed to enhance the robustness of the mediation analyses, future studies with larger samples are needed to confirm the stability of the observed effects (Hayes and Rockwood, 2017). Second, the exclusive use of self-report measures captures participants’ perceived experiences rather than objective indicators; future research would benefit from integrating multiple assessment methods.

Third, the cross-sectional design precludes causal inferences regarding the directionality of the observed relationships. Longitudinal studies are needed to determine whether metacognitive abilities facilitate symptom reduction and subsequent growth, or whether individuals who experience greater posttraumatic growth develop stronger metacognitive skills over time. Another limitation concerns the use of a DSM-IV-based measure (LASC) to assess PTSD symptoms. Although the instrument has demonstrated good psychometric properties, future research should employ DSM-5-based measures, such as the PCL-5 or ITQ, to replicate and extend the present findings (Di Tella et al., 2022; Rossi et al., 2022).

Additionally, the present study did not differentiate among specific components of metacognition. Future research should examine distinct metacognitive domains (e.g., positive vs. negative metacognitive beliefs, metacognitive awareness, control over rumination, and mentalization abilities) to clarify which aspects are most relevant in mediating trauma-related outcomes. Finally, IPV represents a form of prolonged interpersonal trauma. Relational dynamics, persistent perceptions of threat, and loss of autonomy may alter the functioning of metacognitive processes in ways that differ from acute or non-interpersonal trauma. The mediating role of metacognition may vary depending on characteristics of the violence (e.g., severity, duration, and type of IPV), time elapsed since the end of the relationship, perceived safety, and availability of social or institutional support. Individual psychological factors, such as personality traits, coping strategies, and prior trauma exposure, may further influence these processes.

Despite these limitations, the present study provides preliminary evidence supporting the relevance of metacognitive abilities in understanding posttraumatic growth among women survivors of IPV and highlights the need for further research to deepen and refine the investigation of these mechanisms.

## Data Availability

The raw data supporting the conclusions of this article will be made available by the authors, without undue reservation.
